# Prevalence of ligamentous knee injuries in pedestrian versus motor vehicle accidents

**DOI:** 10.1186/s12891-020-03397-w

**Published:** 2020-06-10

**Authors:** R. Garrett Steinmetz, Matthew McDonald, Shaun Tkach, John Hamilton, Gregory Heigle, Kimberly Hollabaugh, David Teague, Douglas Rowles

**Affiliations:** Department of Orthopedic Surgery, University of Oklahoma Health Sciences Center, 800 Stanton L Young Blvd, Oklahoma City, OK 73104 USA

## Abstract

**Background:**

The purpose of this study was to determine the prevalence and characteristics of ligamentous knee injuries and to compare patient demographics, associated injuries and hospital stay to pedestrians who did not sustain a ligamentous knee injury.

**Methods:**

A retrospective review of all adult patients presenting as pedestrians struck by a motor vehicle to a level 1 trauma center over a three-year period was performed. Demographics, length of stay, orthopedic and non-orthopedic traumatic injuries were recorded. Magnetic resonance imaging was reviewed for ligamentous, bony and chondral injuries.

**Results:**

Five hundred thirty-nine patients were included. Sixty-seven (12.4%) patients sustained a total of 84 ligamentous knee injuries. OF these knee injuries that had MRI (55/84), the majority (96%) were multi-ligamentous in nature. Patients with ligamentous knee injury were more likely to also be affected by traumatic brain injury, solid organ injury, cervical and lumbar spine injury, pelvic ring injuries, distal femur fractures, patella fractures, knee dislocations, tibial plateau fractures, tibial pilon fractures, and deep vein thrombosis when compared to patients who did not sustain ligamentous knee injury. Patients who sustained ligamentous knee injury were more likely to require hospital and intensive care admission and had a longer overall hospital stay.

**Conclusion:**

Given the high prevalence of ligamentous knee injuries in this patient population, these patients should be thoroughly evaluated for a ligamentous knee injury. If ligamentous knee injury is suspected, MRI should be considered as a majority of these injuries involved multiple structures. Patients with ligamentous knee injuries often had multi-system injuries with resulting longer hospital stay when compared to those without ligamentous knee injuries.

## Background

Pedestrian versus motor vehicle accidents are a common mechanism of injury in patients presenting to trauma facilities, especially in urban areas. Given the high energy associated with these injuries, the patients often have multiple systems injured, which leads to substantial morbidity and mortality which can lead to longer intensive care and overall hospital stay [1]. Orthopedic injuries are very common and as shown by Brainard et al., lower extremity injuries are the most common orthopedic injuries in patients presenting as pedestrian versus motor vehicle accidents [2]. Reith et al. showed that lower extremity injuries were the most common orthopedic injury and present in 52% of the pedestrian accidents in their review [3]. Peng et al. showed that lower extremity injuries were the most common orthopedic injury and were present in 58.9% of patients who were involved in pedestrian versus motor vehicle accidents [1].

Multiple ligamentous knee injuries are relatively rare, but may be associated with high energy trauma and need to be recognized due to potential devastating complications such as popliteal artery injury, peroneal nerve injury and/or compartment syndrome [4–7]. Previous studies have shown that patients with knee dislocations have a high rate of severe multi-system trauma [4, 8, 9]. Despite this, not much is known about the prevalence of ligamentous knee injuries in this patient population. In a review of 100 multiligamentous knee injuries presenting to a level 1 trauma center, Becker et al. found 23 patients (23%) presented as pedestrians struck by a motor vehicle [4].

The purpose of this study is to determine the prevalence and characteristics of ligamentous knee injuries in patients presenting as pedestrian versus motor vehicle accidents. Another aspect of this study was to compare patient demographics, associated orthopedic and non-orthopedic injuries, intensive care stay and overall hospital stay to patients who did not have ligamentous knee injuries.

## Methods

Institutional Review Board approval was obtained for this retrospective study. All patients greater than 18 years of age involved in pedestrian accidents presenting to a single institution level 1 trauma center from 2013 to 2017 were queried through our institution’s trauma database. Patients who died within 24 h of admission or died prior to orthopedic examination were excluded. Charts were retrospectively reviewed for age at time of injury, gender, height, weight, insurance status, hospital stay, intensive care stay, discharge destination, chest trauma, solid organ injury, vascular injury, facial and skull fractures, traumatic brain injury, gastrointestinal injury, genitourinary injury, spine and spinal cord injury, associated upper and lower extremity injuries. Ligamentous knee injuries were diagnosed either with physical exam or magnetic resonance imaging (MRI). Clinical exam has been shown to reliable for diagnosing anterior cruciate ligamentous, posterior cruciate ligament and posterolateral corner injury [10–13]. For patients with ligamentous knee injuries, charts were reviewed for MRI, specific ligaments or ligamentous complexes injured, meniscal injury, nerve injury, arterial injury, knee dislocation, chondral injury, bone bruise and subchondral fracture. MRI has been shown to be reliable in diagnosing ligamentous injuries about the knee [14–16]. For this study, ligaments included were the anterior cruciate ligament (ACL), posterior cruciate ligament (PCL), medial collateral ligament (MCL) and lateral collateral ligament/posterior lateral corner complex (LCL/PLC). For this study, the LCL/PLC complex was considered to include the lateral collateral ligament, popliteus, popliteofibular ligament, arcuate ligament, fabellofibular ligament, long head of biceps femoris tendon and iliotibial band.

### Statistical analysis

Demographic and clinical characteristics were compared among patients with a ligamentous knee injury and patients without the injury. Chi-square tests were used to compare proportions and a T-test was used to compare continuous data. Wilcoxon rank sum *p*-value was used for length of stay data. The incidence of identified injuries was calculated along with a 95% confidence interval.

## Results

Five hundered and fifty seven patients were identified in our review and 18 patients were excluded due to death within 24 h or death prior to adequate orthopedic evaluation leaving 539 patients to be included in this study. Table 1 shows the demographics of all included patients. Sixty seven patients sustained ligamentous knee injuries giving a prevalence of 12.4% (95% CI: 10–16%). Seventeen patients sustained bilateral ligamentous knee injuries, giving a total of 84 ligamentous knee injuries. There were six knee dislocations, three vascular injuries and three peroneal nerve injuries.

**Table 1:** Patient Demographics and Hospitalization Characteristics

	Overall (***N*** = 539)	No Ligamentous Knee Injury (***N*** = 470)	Ligamentous Knee Injury (***N*** = 69)		
	Count (%)	Count (%)	Count (%)	P-value	OR (95% CI)
Age	43.6	43.7	42.7	0.6425	
Height (cm)	172.9	172.8	173.3	0.6949	
Weight (kg)	82.2	81.8	84.75	.2707	
Body Mass Index (BMI)	27.53	27.41	28.3	.3032	
Sex					
Male	349 (64.7%)	305 (64.9%)	44 (63.8%)	0.9870	0.9 (0.5–1.7)
Female	190 (35.3%)	165 (35.1%)	25 (36.2%)		
Payor status					
Unfunded	211 (39.8%)	184 (39.8%)	27 (39.7%)	0.7380	0.9 (0.7–1.2)
Medicaid	94 (17.7%)	80 (17.3%)	14 (20.6%)		
Medicare	96 (18.1%)	86 (18.6%)	10 (14.7%)		
private ins	126 (23.8%)	110 (23.8%)	16 (23.5%)		
Dept. of Corrections	3 (0.6%)	2 (0.4%)	1 (1.5%)		
Hospital admission					
No	119 (22.1%)	115 (24.5%)	4 (5.8%)	0.0005	7.0 (2.1–22.5)
Yes	420 (77.9%)	355 (75.5%)	65 (94.2%)		
ICU care					
No	314 (58.3%)	285 (60.6%)	29 (42.0%)	0.0034	2.1 (1.3–3.6)
Yes	225 (41.7%)	185 (39.4%)	40 (58.0%)		
Discharge Disposition					
Home	338 (62.7%)	306 (65.1%)	32 (46.4%)	0.0546	0.8 (0.7–1.0)
Long Term Acute Care Facility	28 (5.2%)	22 (4.7%)	6 (8.7%)		
Skilled Nursing Facility	21 (3.9%)	16 (3.4%)	5 (7.2%)		
Inpatient rehab	109 (20.2%)	89 (18.9%)	20 (29.0%)		
Hospital	17 (3.1%)	16 (3.3%)	1 (1.4%)		
Dept. of Correction	4 (.7%)	3 (.6%)	1 (1.4%)		
Death	22 (4.1%)	18 (3.8%)	4 (5.8%)		

MRI was available for 55/84 injured knees, the most common ligaments injured were anterior cruciate ligament (47), medial collateral ligament (45), lateral collateral ligament/ posterior lateral corner ligamentous complex (44) and the posterior cruciate ligament (38). Additionally, on MRI there were 37 instances of bone bruises or subchondral impaction fractures involving either the medial or lateral femoral condyles and/or medial or lateral tibial plateaus. Forty-seven meniscal injuries (22 medial and 25 lateral) and 9 acute chondral injuries. Table 2 shows the most common patterns of ligamentous injuries.

**Table 2:** Ligamentous Pattern of Injury Based on Magnetic Resonance Imaging

LIGAMENTOUS INJURY PATTERN	
ACL + PCL + MCL + LCL/PLC	23
ACL + MCL + LCL/PLC	7
ACL + PCL + MCL	6
ACL + PCL + LCL/PLC	4
ALC + MCL	3
MCL + LCL/PLC	3
ACL + LCL/PLC	2
PCL + MCL + LCL/PLC	2
PCL + LCL/PLC	2
ACL + PCL	1
ACL	1
MCL	1
	*n* = 55

Patients who sustained ligamentous knee injuries were significantly more likely to have associated traumatic brain injury, solid organ injury and deep vein thrombosis of the lower extremity (Table 3). As far as associated orthopedic injuries, patients who had ligamentous knee injuries were more likely to have cervical spine injury, lumbar spine injury, pelvic ring injuries, acetabular fractures, distal femur fractures, patella fractures, tibial plateau fractures, tibial pilon fractures and knee dislocations when compared to those who did not have ligamentous knee injuries (Table 4).

**Table 3:** Non Orthopedic Injuries in Ligamentous and Non Ligamentous Knee Injury Patients

	Overall (N = 539)	No Lig Knee Injury (***N*** = 472)	Lig Knee Injury (***N*** = 67)		
	Count (%)	Count (%)	Count (%)	P-value	OR (95%CI)
Spinal cord injury (SCI)					
No	526 (97.6%)	459 (97.2%)	67 (100.0%)		
Yes	13 (2.4%)	13 (2.8%)	0 (0.0%)	0.1686	0.97 (0.95–0.98)
Traumatic brain injury (TBI)					
No	420 (77.9%)	375 (79.4%)	45 (67.2%)		
Yes	119 (22.1%)	97 (20.6%)	22 (32.8%)	**0.0233**	**1.9 (1.1–3.3)**
Face/skull fracture					
No	395 (73.3%)	351 (74.4%)	44 (65.7%)		
Yes	144 (26.7%)	121 (25.6%)	23 (34.3%)	0.1324	1.5 (0.9–2.6)
Chest Trauma					
No	333 (61.8%)	297 (62.9%)	36 (53.7%)		
Yes	206 (38.2%)	175 (37.1%)	31 (46.3%)	0.1473	1.5 (0.9–2.5)
Solid organ injury					
No	448 (83.1%)	400 (84.7%)	48 (71.6%)		
Yes	91 (16.9%)	72 (15.3%)	19 (28.4%)	**0.0074**	**2.2 (1.2–4.0)**
Gastrointestinal I injury					
No	521 (96.7%)	457 (96.8%)	64 (95.5%)		
Yes	18 (3.3%)	15 (3.2%)	3 (4.5%)	0.5795	1.4 (0.4–5.1)
Genitourinary injury					
No	522 (96.8%)	458 (97.0%)	64 (95.5%)		
Yes	17 (3.2%)	14 (3.0%)	3 (4.5%)	0.5121	1.5 (0.4–5.5)
Vascular injury					
No	494 (91.7%)	433 (91.7%)	61 (91.0%)		
Yes	45 (8.3%)	39 (8.3%)	6 (9.0%)	0.8519	1.1 (0.4–2.7)
Deep vein thrombosis					
No	490 (90.9%)	434 (91.9%)	56 (83.6%)		
Yes	49 (9.1%)	38 (8.1%)	11 (16.4%)	**0.0258**	**2.2 (1.1–4.6)**

**Table 4:** Orthopedic Injuries in Ligamentous and Non Ligamentous Knee Injury Patients

	Overall (N = 539)	No Ligamentous Knee Injury (N = 472)	Ligamentous Knee Injury (N = 67)		
	Count (%)	Count (%)	Count (%)	P-value	OR (95% CI)
Cervical Spine Injury					
No	464 (86.1%)	412 (87.3%)	52 (77.6%)		
Yes	75 (14.2%)	60 (12.7%)	15 (22.4%)	**0.0335**	**2.0 (1.1–3.7)**
Thoracic Spine Injury					
No	474 (87.8%)	418 (88.6%)	56 (83.6%)		
Yes	65 (12.2%)	54 (11.7%)	11 (15.9%)	0.2445	1.5 (0.8–3.1)
Lumbar Spine Injury					
No	456 (84.6%)	407 (86.2%)	49 (73.1%)		
Yes	83 (15.4%)	65 (13.8%)	18 (26.1%)	**0.0056**	**2.3 (1.3–4.2)**
Upper Extremity Fracture					
No	383 (71.1%)	335 (71.0%)	48 (71.6%)		
Yes	156 (28.9%)	137 (29.0%)	19 (28.4%)	0.9103	0.96 (0.5–1.7)
Pelvic Ring Injury					
No	408 (75.7%)	365 (77.3%)	43 (64.2%)		
Yes	131 (24.3%)	107 (22.7%)	24 (35.8%)	**0.0188**	**1.9 (1.1–3.3)**
Acetabular Fracture					
No	492 (91.3%)	437 (92.6%)	55 (82.1%)		
Yes	47 (8.7%)	35 (7.4%)	12 (17.9%)	**0.0044**	**2.7 (1.3–5.6)**
Proximal Femur Fracture					
No	509 (94.4%)	448 (94.9%)	61 (91.0%)		
Yes	30 (5.6%)	24 (5.1%)	6 (9.0%)	0.1960	1.8 (0.7–4.7)
Diaphyseal Femur Fracture					
No	512 (95.0%)	448 (94.9%)	64 (95.5%)		
Yes	27 (5.0%)	24 (5.1%)	3 (4.5%)	0.8312	1.9 (0.9–3.8)
Distal Femur Fracture					
No	532 (98.7%)	468 (99.2%)	64 (95.5%)		
Yes	7 (1.3%)	4 (0.8%)	3 (4.5%)	**0.0141**	**5.5 (1.2–25.1)**
Patellar Fracture					
No	531 (98.5%)	467 (98.9%)	64 (95.5%)		
Yes	8 (1.5%)	5 (1.1%)	3 (4.5%)	**0.0304**	**4.4 (1.0–18.8)**
Quadriceps tendon injury					
No	538 (99.8%)	471 (99.8%)	67 (100.0%)		
Yes	1 (0.2%)	1 (0.2%)	0 (0.0%)	0.7061	0.99 (0.99–1.1)
Patellar tendon injury					
No	537 (99.6%)	471 (99.8%)	66 (98.5%)		
Yes	2 (0.4%)	1 (0.2%)	1 (1.5%)	0.1067	7.1 (0.4–115.4)
Tibial Plateau Fracture					
No	491 (91.1%)	447 (94.7%)	44 (65.7%)		
Yes	48 (8.9%)	25 (5.3%)	23 (34.3%)	**<.0001**	**4.1 (2.4 - 7.1)**
Tibial shaft fracture					
No	483 (89.6%)	423 (89.6%)	60 (89.6%)		
Yes	56 (10.4%)	49 (10.4%)	7 (10.4%)	0.9867	1.0 (0.4–2.3)
Tibial pilon fracture					
No	530 (98.3%)	467 (98.9%)	63 (94.0%)		
Yes	9 (1.7%)	5 (1.1%)	4 (6.0%)	**0.0033**	**5.9 (1.6–22.7)**
Ankle Fracture +/− Dislocation					
No	491 (91.1%)	428 (90.7%)	63 (94.0%)		
Yes	48 (8.9%)	44 (9.3%)	4 (6.0%)	0.3674	0.6 (0.2–1.8)
Hindfoot Fracture +/− Dislocation					
No	527 (97.8%)	461 (97.7%)	66 (98.5%)		
Yes	12 (2.2%)	11 (2.3%)	1 (1.5%)	0.6635	0.6 (0.1–5.00
Midfoot Fracture +/− Dislocation					
No	527 (97.8%)	460 (97.5%)	67 (100.0%)		
Yes	12 (2.2%)	12 (2.5%)	0 (0.0%)	0.1869	0.97 (0.96–0.99)
Forefoot Fracture +/− Dislocation					
No	514 (95.4%)	453 (96.0%)	61 (91.0%)		
Yes	25 (4.6%)	19 (4.0%)	6 (9.0%)	0.0726	2.3 (0.9–6.1)
Hip dislocation					
No	529 (98.1%)	464 (98.3%)	65 (97.0%)		
Yes	10 (1.9%)	8 (1.7%)	2 (3.0%)	0.4640	1.8 (0.4–8.6)
Knee dislocation					
No	532 (98.7%)	470 (99.6%)	62 (92.5%)		
Yes	7 (1.3%)	2 (0.4%)	5 (7.5%)	**<.0001**	**19.0 (3.6–99.1)**

Tables 1 and 5 shows hospital and intensive care stay characteristics. Patients with ligamentous knee injuries were more likely to be admitted to the hospital and to the intensive care unit and had significantly longer hospital stay compared to those who did not have ligamentous knee injuries.

**Table 5:** Length of Hospital and Intensive Care Stay

	No Ligamentous Knee Injury		Ligamentous Knee Injury		
	N	Median Length of Stay	N	Median Length of Stay	P-value
Length of Hospital Stay	470	5 days (Range 0–147)	69	11 days (Range 0–74)	< .0001
Length of Intensive Care Stay	186	5 days (Range 1–60)	39	8 days (Range 1–60)_	.12

The preferred treatment algorithm for patients was to evaluate the stability of the knee while in the hospital and if grossly unstable, knee spanning external fixator was indicated. If the knee remained reduced, then the patient was placed into a hinged knee brace while in the hospital. The patients were typically seen in clinic after discharge from the hospital and in patients with ligamentous knee injuries that resulted in knee instability, ligamentous reconstruction was performed as early as possible, as has been suggested by Levy et al. [17]

## Discussion

Pedestrian versus motor vehicle accidents represent a high-energy mechanism that is common in urban areas and is associated with substantial morbidity and mortality [1, 18]. Over a 10 year period (2006–2015) Chong et al. showed there were 47,789 deaths and 674,414 injuries [18]. Peng et al. reviewed 5000 pedestrians injured over a 3 year period by accessing a county database [1]. Their review showed that most common injuries included musculoskeletal (34.3%), head and neck (30.0%), external (24.4%), abdomen and pelvis (3.9%), chest (2.4%) and spine (1.8%). Additionally, they showed that at the time of discharge, 78% of patients had a temporary disability, 4% had a permanent handicap, and only 16% were functioning at preadmission capacity [1]. In another large review of 4435 patients, Reith et al. compared pedestrian versus motor vehicle patients to patients presenting as motor vehicle occupants [3]. They showed that the pedestrian cohort was more severely injured and had a higher rate of head injuries, pelvis injuries and lower extremity injuries [3]. Previous studies have shown that patients with traumatic knee dislocations and multiligamentous knee injuries have a high rate of associated multi-system trauma which can be life-threatening [4, 8, 9]. In our review, patients who sustained a ligamentous knee injury were statistically more likely to sustain traumatic brain injury and solid organ injury. Additionally, cervical spine injury, lumbar spine injury, pelvic ring injuries, acetabular fractures, distal femur fractures, patella fractures, tibial plateau fractures, tibial pilon fractures and knee dislocations were statistically more likely to occur in those with ligamentous knee injuries. Lower extremity DVT was more common in patients who sustained ligamentous knee injury as well. These co-existing injuries should be acknowledged by the treating physician and could complicate the management of the ligamentous knee injury.

Orthopedic injuries, especially of the lower extremity, are very common in the pedestrian versus motor vehicle accident population. In one of the largest reviews of orthopedic trauma in this population, Brainard et al. showed 96 injuries of the lower extremities in 115 patients who presented with this mechanism of injury [2]. Despite this, there has been little literature which looks specifically at ligamentous knee injuries in the pedestrian versus motor vehicle patient population. Our review shows a prevalence of 12.4% (95% CI: 10–16%) for ligamentous knee injuries in the pedestrian versus motor vehicle population. Becker et al. reviewed 100 multiligamentous knee injuries that presented to a level 1 trauma center, and found 23 patients (23%) presented as pedestrians struck by a motor vehicle [4]. Moatshe et al. evaluated 303 patients with a knee dislocation and showed that 5.9% of patients were pedestrians struck by a motor vehicle [19]. Ferry et al. reviewed all patients who had a knee injury over a 14 year period and found that 14% of these patients were pedestrians who were struck by motor vehicles [20]. Forward et al. showed that nearly half of the patients in their cohort sustained a knee injury following pedestrian versus motor vehicle accidents and 22% of those knee injuries were ligamentous in nature [21]. It can be difficult to diagnose a ligamentous knee injury in this patient population given that they often have injuries of multiple systems, thus a high index of suspicion is necessary. We recommend performing a thorough ligamentous knee exam in patients presenting with this mechanism. Not all patients in our review received MRI, however, in those that did, the most common pattern of injury was ACL + PCL + MCL + LCL/PLC complex and all but two were multi-ligamentous in nature. Additionally, we had a large number of concomitant intra-articular injuries which were identified on MRI. Given that a majority of these are multi-ligamentous, we recommend obtaining MRI early in the hospital stay in patients with suspected ligamentous knee injury who are stable enough to have the study and would benefit from potential repair or reconstruction. Although not specifically reviewed in this study, our recommendation for managing these injuries is early operative intervention as described by Levy et al. [17]

Previous studies have reported on length of intensive care and hospital stay following pedestrian versus motor vehicle accidents. Peng et al. showed that intensive care 6.5 ± 0.3 days and overall hospital stay was 7.4 ± 0.2 days [1]. In their comparison to patients who were occupants of motor vehicle accidents, Reith et al. showed that pedestrians had a similar length of intensive care and overall hospital stay [3]. McElroy et al. showed that pedestrian versus motor vehicle victims had an average hospital stay of 10.8 days and intensive care stay of 6 ± 7.5 days [22]. Specifically looking at knee injuries, Georgiadis et al. showed that the average length of hospital stay in trauma patients who sustained a knee dislocation was 11.4 days [23]. Woodmass et showed that the average ICU stay in patients who sustained multi-ligamentous knee injuries was 2.8 days [24]. Additionally, they showed that 48% of patients with multi-ligamentous knee injuries required intensive care stay [24]. In our review, pedestrians who sustained a ligamentous knee injury had a longer overall hospital stay (11 days versus 5 days) and were more likely to need inpatient admission and intensive care stay when compared to those who did not sustain a ligamentous knee injury.

This study does have limitations which need to be mentioned. This is a retrospective study and thus subject to selection bias. For this review the clinical follow up was very poor, therefore there could have been patients who were lost to follow up which sustained a ligamentous knee injury that was missed while inpatient. Additionally, patients were excluded if they died within 24 h or died prior to orthopedic evaluation. These patients were often severely injured and potentially could have had ligamentous knee injuries as well. Given the poor follow up, we chose not to investigate how these patients fared after surgical or non-surgical management, thus this is a practice gap which could be evaluated with future studies. MRI was not obtained in all patients who sustained a ligamentous knee injury, typically this was due to patients who were clinically too unstable or if the knee injury was managed conservatively by the attending surgeons.

## Conclusion

In conclusion, patients presenting as pedestrian versus motor vehicle have a high prevalence of ligamentous knee injuries and thus a high index of suspicion is necessary when evaluating these patients. Pedestrian versus motor vehicle accidents with ligamentous knee injuries were more likely to have multiple orthopedic and non-orthopedic injuries when compared to those without ligamentous knee injuries, thus representing the high energy of this mechanism. Patients sustaining ligamentous knee injuries were also more likely to be admitted to the hospital and intensive care unit and the length of overall hospital stay were also longer when compared to those who did not sustain ligamentous knee injury.

## Data Availability

The datasets during and/or analyzed during the current study available from the corresponding author on reasonable request.

## Abbreviations

ACL: Anterior Cruciate Ligament
PCL: Posterior Cruciate Ligament
MCL: Medial Collateral Ligament
LCL: Lateral Collateral Ligament
PLC: Posterior Lateral Corner)
